# Establishment of an in vitro choroid complex system for vascular response screening

**DOI:** 10.1038/s41598-024-67069-8

**Published:** 2024-07-12

**Authors:** Heonuk Jeong, Deokho Lee, Kazuno Negishi, Kazuo Tsubota, Toshihide Kurihara

**Affiliations:** 1 Department of Ophthalmology, Keio University School of Medicine, 35 Shinanomachi, Shinjuku-Ku, Tokyo 160-8582 Japan; 2 Laboratory of Photobiology, Keio University School of Medicine, 35 Shinanomachi, Shinjuku-Ku, Tokyo 160-8582 Japan; 3 Tsubota Laboratory, Inc, 304 Toshin Shinanomachi-Ekimae Bldg., 34 Shinanomachi, Shinjuku-Ku, Tokyo 160-0016 Japan

**Keywords:** Choroid, In vitro culture system, Fibroblast, Endothelial cell, Vasoregulation, Tissue engineering, Experimental models of disease

## Abstract

The choroid, a vascularized tissue situated between the retina and the sclera, plays a crucial role in maintaining ocular homeostasis. Despite its significance, research on choroidal abnormalities and the establishment of effective in vitro models have been limited. In this study, we developed an in vitro choroid model through the co-culture of human induced pluripotent stem cells (hiPSC)-derived endothelial cells (ECs) and mouse choroidal fibroblasts (msCFs) with hiPSC-derived retinal pigment epithelial (RPE) cells via a permeable membrane. This model, inclusive of ECs, CFs, and RPE cells, exhibited similarities with in vivo choroidal vessels, as confirmed through immunohistochemistry of extracellular matrix markers and vascular-related markers, as well as choroid angiogenesis sprouting assay analysis. The effectiveness of our in vitro model was demonstrated in assessing vascular changes induced by drugs targeting vasoregulation. Our model offers a valuable tool for gaining insights into the pathological mechanisms underlying choroid development and the progression of choroidal vascular diseases.

## Introduction

The choroid is the posterior part of uvea in the eye, which is located between the sclera and the retinal pigment epithelium (RPE). Four different layers (Bruch’s membrane, choriocapillaris (CC), Sattler’s layer, and Haller’s layer) exist in the choroid^1^. As a highly vascularized tissue, the choroid plays a crucial functional role by providing abundant oxygen and nutrients to the RPE and neuronal cells in the retina as well as sclera, the outer ocular tissue, while also facilitating the removal of waste products from these cells^1^. Additionally, it contributes to the posterior ocular tissue, the sclera^2^. Choroidal abnormalities can cause various ocular diseases especially, age-related macular degeneration (AMD)^3–5^. In terms of the ocular development, the choroid plays a vital role in regulating scleral remodeling and eye growth^2,6–8^. Importantly, it enables choroidal accommodation, which adapts the thickness of the choroid to facilitate clear focusing on an image when the retina detects any signs of defocus^9–11^. Clinical research has indicated a notable reduction in choroidal thickness among individuals with high myopia, with subfoveal choroidal thickness displaying a negative correlation with axial length^3,12,13^. Consequently, an expanding body of scientific experts is considering choroidal thickness as a potential clinical biomarker for both the initiation and advancement of myopia^14,15^. In this regard, pathologic myopia can be caused by altered choroidal vasculature.

To understand the development and progression of choroidal vascular diseases, researchers have been using various experimental in vitro choroid models created through techniques such as 3D bioprinting and polymer synthesis ^16–19^. However, targeting the choroid tissue in vivo still faces limitations and requires further investigations, as co-culture systems are essential due to the presence of various cell types necessary for maintaining its function. In our current study, we aimed to set-up the novel co-culture system in vitro to mimic the in vivo choroidal vasculature by combining human induced pluripotent stem cells (hiPSC)-derived endothelial cells (ECs), mouse choroidal fibroblasts (msCFs), and hiPSC-derived RPE cells. This choroid in vitro model was utilized to evaluate vascular responses to commercially available drugs, assessing vasoconstriction and vasodilation effects. Our in vitro model described here is anticipated to offer a straightforward approach to understanding the pathological mechanisms underlying choroid development and to screening promising novel drug candidates for the treatment of choroidal vascular diseases.

## Results

### Construction of the choroid in vitro models and extracellular matrix composition

**Figure 1:**
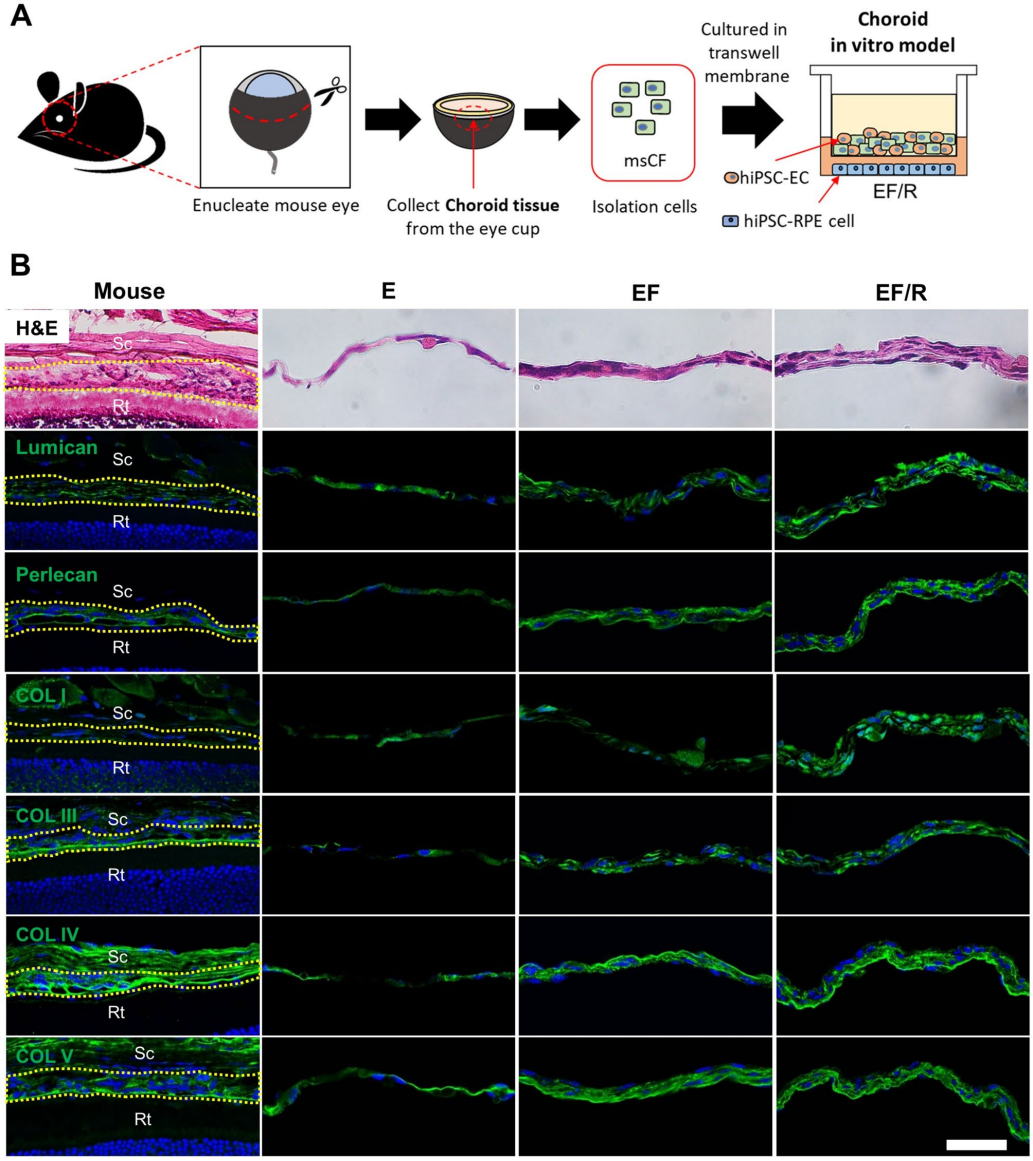
Choroid-like extracellular matrix in the co-culture model. (A) Construction of choroid in vitro model. Mouse choroidal fibroblasts (msCFs) isolated from C57BL/6 J mouse eyes and human induced pluripotent stem cells (hiPSC)-derived endothelial cells (EC) were cultured on the transwell membrane with hiPSC-derived retinal pigment epithelial (RPE) cells on the bottom of the plate. (B) Representative images of H&E staining and the expression of lumican, perlecan, collagen I, III, IV, and V in the mouse native choroid (Mouse) and the choroid in vitro models (E, EF, and EF/R) cultured for one week. Immunostaining appears in green and nuclei were counterstained with DAPI (blue). The mouse choroid is outlined by a yellow dashed line. Scale bar: 50 µm. Sc, Sclera; Rt, Retina.

The choroid in vitro model was generated with the combinations of three cell types, msCFs isolated from C57BL6J mice, hiPSC-derived ECs, and hiPSC-derived RPE cells (Fig. 1A). "E" represents the EC-only without RPE cells at the bottom, "E/R" is the EC-only with RPE cells at the bottom, "EF" is the mixed model of ECs and CFs without RPE cells at the bottom, and "EF/R" is the mixed model with both ECs and CFs and RPE cells at the bottom.

We first characterized our in vitro choroid models (E, EF, and EF/R) in comparison with the choroid in BALB/c mice, using immunohistochemistry as well as H&E staining (Fig. 1B). One week after cell culture, morphology of EF/R showed multi-layers as similar as that of the choroid in vivo. Choroidal morphology in the EF group was also similar to that in vivo, while E group could not be well-formed, which showed only one layer of cells. Lumican and perlecan, the extracellular matrix proteoglycans in the choroid^17,20^, were well-expressed in the EF and EF/R groups, compared with the mouse choroid.

Next, we examined whether the collagen (COL) family could be well-expressed in our in vitro model. COL I, III, IV, and V expressions were clearly detected in the EF and EF/R groups, similar to those in the mouse choroid. However, the E group had weak expressions in the collagen family, implying that CFs might be necessary to establish the choroid-like extracellular matrix.

### Features of vessels in the choroid in vitro

**Figure 2:**
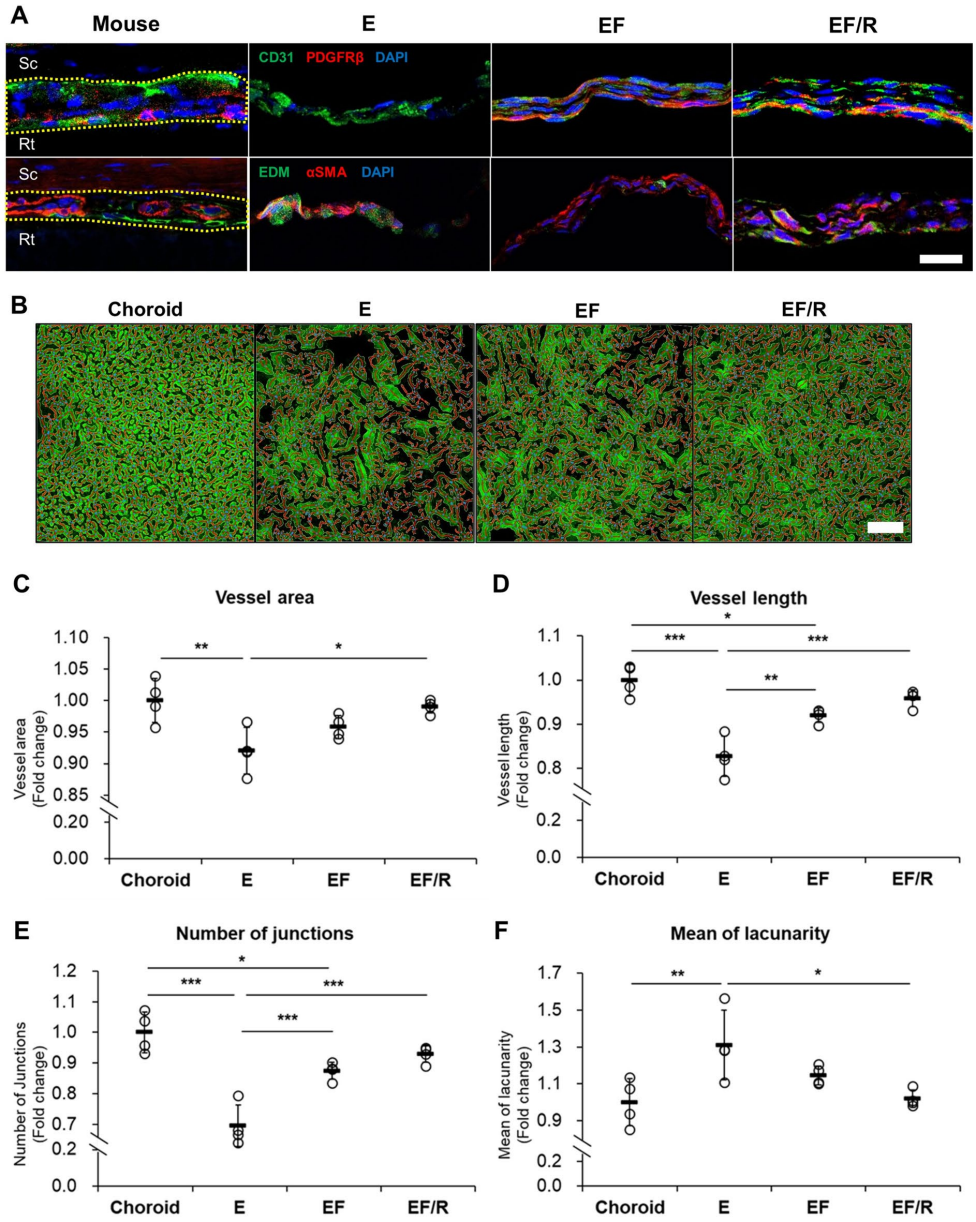
Vasculature network formation in the co-culture model. (**A**) Representative images of immunostaining with CD31 (green) and PDGFRβ (red) in the upper panels and with endomucin (EDM, green), αSMA (red) in the lower panels in the mouse native choroid (Mouse) and the choroid in vitro models (E, EF, and EF/R) after one week of culture. Nuclei were counterstained with DAPI (blue). The mouse choroid is outlined by a yellow dashed line. Scale bar: 50 µm. Sc, Sclera; Rt, Retina. (B-F) Quantification of vessel formation in the constructed choroid models compared to the mouse choroid reveals that the EF/R group is the closest match to the mouse choroid. Segmented and skeletonized immunofluorescence images (**B**) and analysis of vessel area (**C**), vessel length (**D**), number of junctions (**E**), and mean of lacunarity (**F**) using AngioTool. The quantification data was normalized by the values of the mouse choroid. Scale bar: 100 µm. n = 4. * p < 0.05, ** p < 0.01, *** p < 0.001, One-way ANOVA with a post-hoc Tukey’s test.

As the choroid is a layer of blood vessels, we evaluated features of the choroidal vasculature in our current system using immunofluorescence staining with a CD31 antibody for endothelial cells, a platelet-derived growth factor receptor beta (PDGFRβ) antibody for pericytes, an endomucin antibody for CC, and an alpha-smooth muscle actin (αSMA) antibody for smooth muscle cells (Fig. 2A). We found that only CD31 was expressed in the E group. While PDGFRβ and CD31 expressions were detected in the EF and EF/R groups, EF/R was relatively more similar with the choroid in vivo, displaying separated layers. Furthermore, we further characterized our in vitro models with αSMA and endomucin antibodies. Similar to the choroid in vivo, the lower side of the EF/R group showed a strong expression of endomucin, indicating CC-like vasculature.

Next, the vessel network in our choroid models was evaluated using AngioTool, a software designed for the quantitative analysis of vasculature (Fig. 2B–F). The EF/R group demonstrated values closest to those of the mouse choroid. Specifically, the EF/R group exhibited the highest vessel area, number of junctions, and vessel length among the other groups, while the E group showed the lowest values. In terms of lacunarity, which indicates the gaps between the vessels^21^, the EF/R group had the lowest value, with the E group showing the highest. Among the groups, the values generally followed the order of E, EF, and EF/R. To examine the influence of CFs in the presence of RPE cells, we compared the models with and without co-culture of CFs (Supplementary Fig. S1). EF/R showed higher vessel area, vessel length, and number of junctions, and lower in lacunarity than the model without CFs (E/R), indicating differences between the presence and absence of CFs.

### In vitro choroid angiogenesis sprouting assay

**Figure 3:**
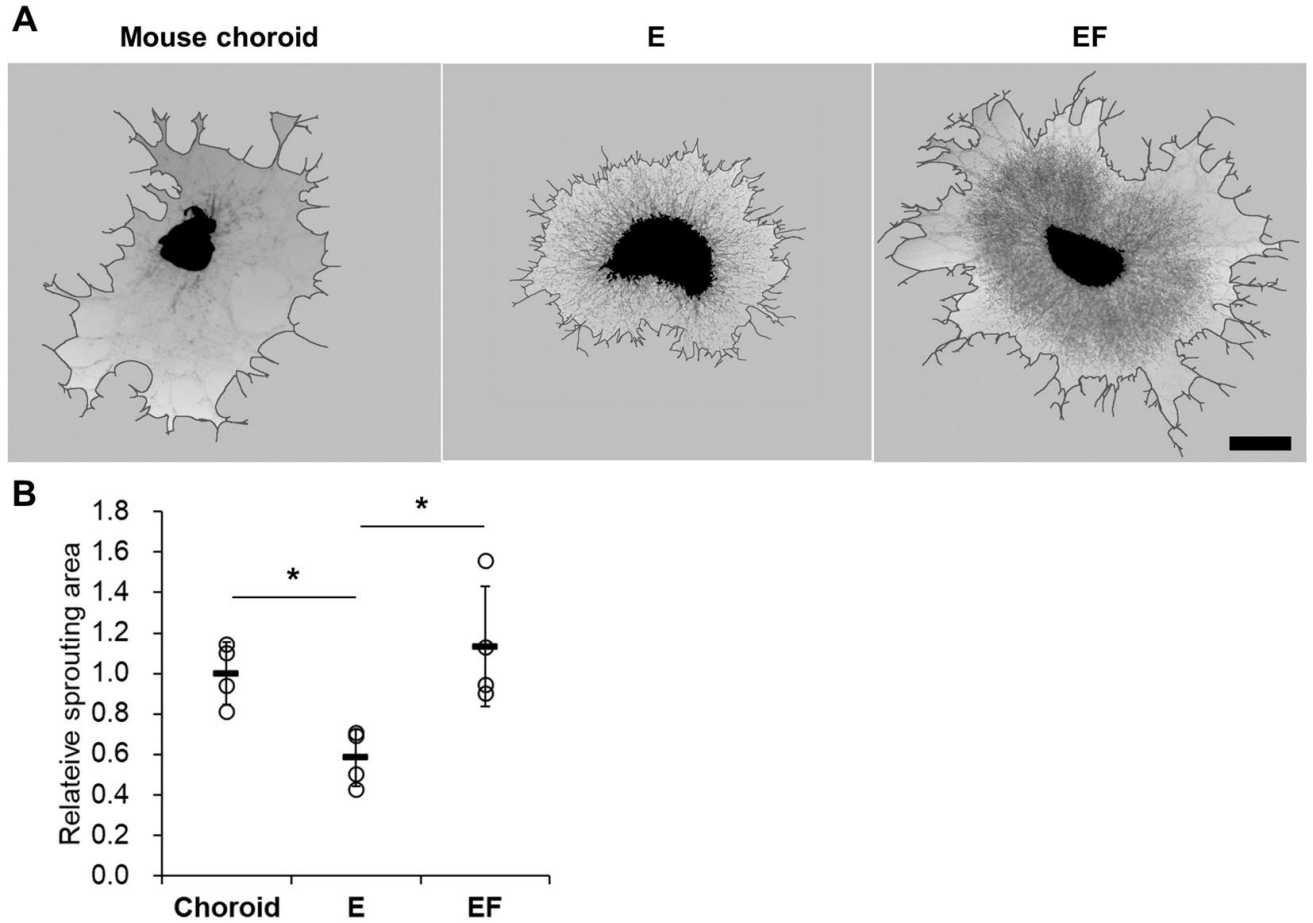
Choroid sprouting assay for angiogenesis of the constructed choroid models. (**A**) Representative images of choroid sprouts after 5 days of culture in the choroid of the C57BL/6 J mouse, E, and EF groups, Scale bar: 1 mm. (**B**) Computed sprouting area normalized by that of C57BL/6 J mouse choroid. Compared to the mouse choroid, the EF group shows more similar sprouting area than the E group. n = 4. * p < 0.05, One-way ANOVA with a post-hoc Tukey’s test.

A choroidal sprouting assay ex vivo has generally been used to complement in vivo studies of microvascular behavior by quantifying sprouting area^22^. We applied this assay to our system to assess its suitability for in vitro models (Fig. 3). We compared microvascular angiogenesis in our models depending on the presence or absence of msCFs. We found that the EF group had sprouting areas comparable to the mouse choroid, while the E group exhibited smaller areas.

### Gene profiling in the choroid in vitro

**Figure 4:**
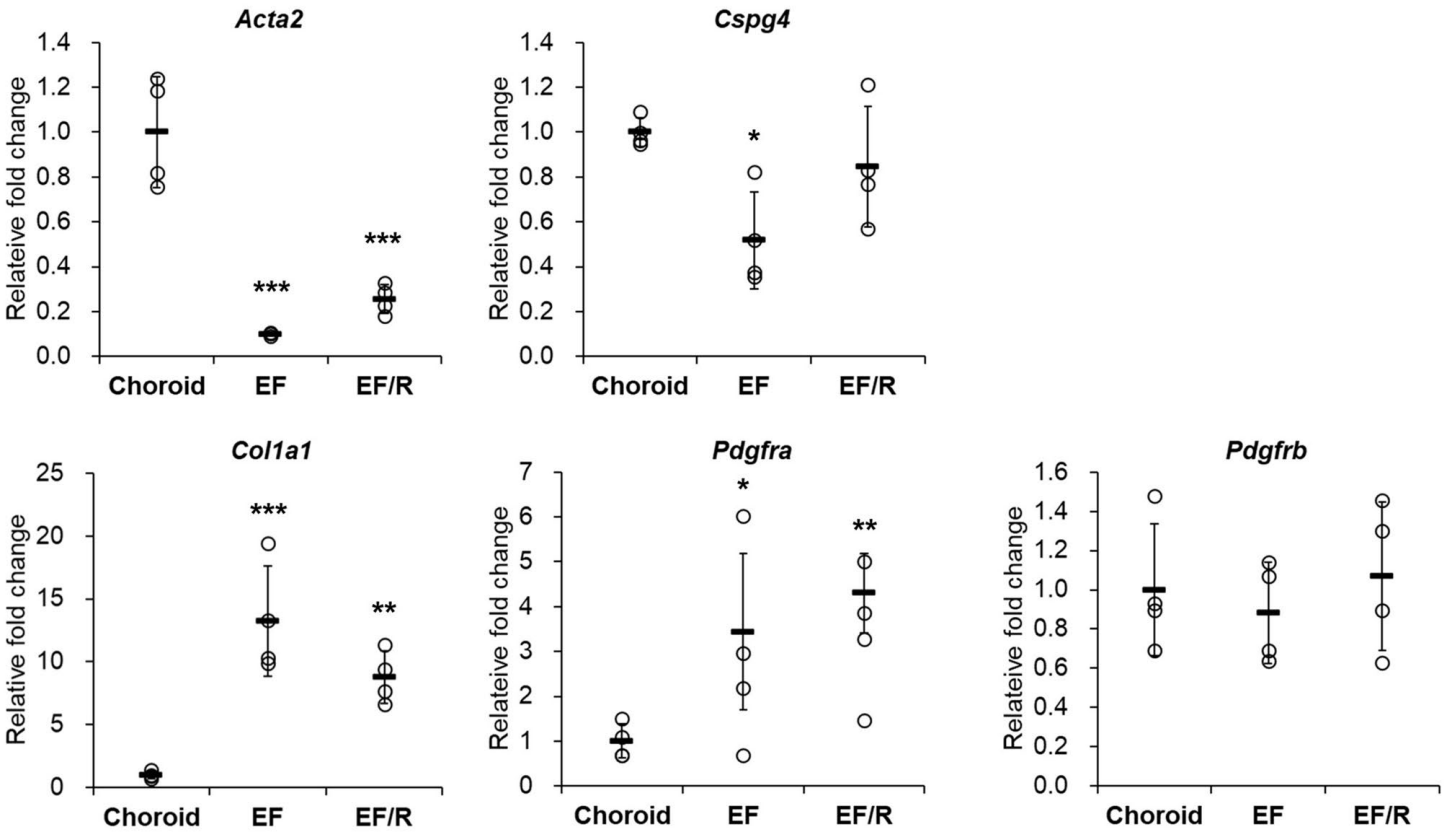
Gene profiling of smooth muscle cell markers (*Acta2* and *Cspg4*) and stromal cell markers (*Pdgfra*, *Pdgfrb*, and *Col1a1*) in the constructed in vitro models (EF and EF/R) and the mouse choroid demonstrated the functional role of choroid-derived fibroblasts in choroid tissue construction. n = 4. * p < 0.05, ** p < 0.01, *** p < 0.001 compared to the mouse choroid, One-way ANOVA with a post-hoc Tukey’s test.

For gene profiling of the choroid in our in vitro models constructed by mouse choroid-derived fibroblasts, we compared the EF and EF/R groups with the mouse choroid, examining stromal cell markers (*Pdgfra*, *Pdgfrb*, and *Col1a1*) and smooth muscle cell markers (*Acta2* and *Cspg4*) (Fig. 4). We observed that *Col1a1* and *Pdgfra* mRNA expressions were higher in the EF and EF/R groups than those in the mouse choroid, whereas *Pdgfrb* mRNA expression was not significantly different among all groups. In terms of smooth muscle cell genes, *Acta2* and *Cspg4* expressions in our in vitro models were relatively lower than those in the mouse choroid, suggesting that choroid-derived fibroblasts might function as stromal cells in constructing the choroid tissue in vitro.

### Drug Interventions using the Choroid in vitro

**Figure 5:**
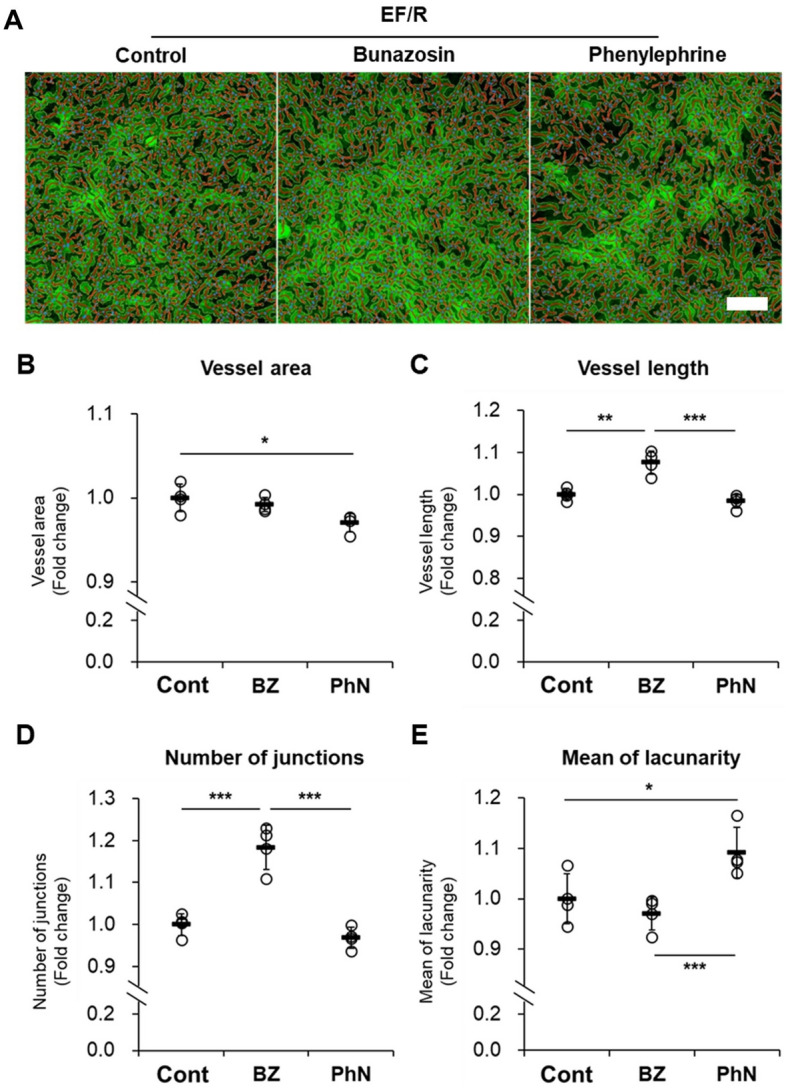
Quantification of vessel formation in the constructed in vitro model (EF/R) responds vasodilator (10 µM bunazosin, BZ) and vasoconstrictor (10 µM phenylephrine, PhN). (**A**) Segmented and skeletonized immunofluorescence images after drug treatment (A) and analysis of vessel area (**B**), vessel length (**C**), number of junctions (**D**), and mean of lacunarity (**E**) using AngioTool. The quantification data was normalized by the values of the non-treated group (Cont). Scale bar: 100 µm. n = 4. * p < 0.05, ** p < 0.01, *** p < 0.001, One-way ANOVA with a post-hoc Tukey’s test.

Our previous research^23^ and that of Zhou X et al.^24^ have reported that choroidal blood perfusion can be pharmacologically regulated by an alpha-1 adrenergic antagonist (bunazosin) or an agonist (phenylephrine). We recently reported that the application of bunazosin could inhibit myopia progression by increasing blood flow in choroidal blood vessels in myopia-induced mice^23^. Conversely, Zhou X et al. have reported that the application of phenylephrine could promote myopia progression by decreasing blood flow in guinea pigs^24^. These findings suggest that choroidal vasoregulation might be an important factor in controlling myopia progression. Therefore, we used bunazosin or phenylephrine to investigate whether the desired vasodilation or vasoconstriction effect of each drug, as observed in the in vivo experimental results of previous studies, is reproducible in our in vitro model. 5 h after drug treatment to EF/R, vascular factors were evaluated (Fig. 5). Compared to the control group (EF/R without drug treatment), vessel area, number of junctions, vessel length, or mean of lacunarity was changed after treatment with bunazosin or phenylephrine. The total number of junctions and vessel length increased in the bunazosin-treated group. In the phenylephrine-treated group, vessel area decreased, and mean of lacunarity increased as the non-vessel area expanded due to its vasoconstriction effect.

## Discussion

In this study, we characterized our in vitro choroid model, developed by co-culturing of hiPSC-ECs with msCFs and hiPSC-RPE cells, utilizing various cellular markers and functional vascular assays. To better mimic the in vivo choroidal condition, the co-culture of various cell types becomes imperative, as demonstrated by our histological data showcasing lumican, perlecan, COL I, COL III, COL IV, and COL V-positive tissue morphologies. Many types of collagens and proteoglycans have been reported to be expressed in the native choroid^17,20,25^. In particular, lumican and perlecan are more highly expressed among proteoglycans^26^.

Furthermore, the inclusion of msCFs and RPE cells was a pivotal factor in mimicking the layered structure of the choroid in vivo. This is evident in tissue morphologies positive for PDGFRβ (pericytes), CD31 (ECs), αSMA (stromal cells), and endomucin (CC). Our vessel network analyses revealed significant differences between our in vitro co-culture choroid model (EF/R) and the EC-only group (E), while similarities were observed between EF/R and the native choroid. The construction of a choroid-like tissue by choroidal fibroblasts, influenced by the RPE-cultured supernatant supplied through the membrane of the transwell insert, enhanced its resemblance to the in vivo choroid. The in vitro RPE-cultured supernatant, known to contain growth factors such as vascular endothelial growth factor (VEGF) associated with choroidal EC development^16,18,27,28^, might play a crucial role in this model. In the EF group of this study, a layer resembling the CC was not visibly present in endomucin immunostaining. Our previous studies demonstrated the absence of CC in RPE-specific VEGF conditional knockout mice, emphasizing the significance of VEGF in CC development and maintenance^8,29^. This emphasizes the essential role of co-culture for an accurate representation of the choroid in vivo. This insight should be carefully considered to develop robust in vitro choroid models.

Lehmann GL et al. conducted a single-cell RNA sequencing, revealing that mouse choroidal stromal cells might exhibit high expression levels of *Col1a1*, *Pdgfra*, and *Pdgfrb*^30^. In our constructed models, we also observed elevated levels of *Col1a1* and *Pdgfra*, indicating the crucial role of msCFs in choroidal stroma construction. The expression level of *Pdgfrb*, a known pericyte marker^31,32^, was comparable between our models and native tissue. Particularly in EF/R, PDGFRβ expression was observed in the choriocapillaris-like layer. This aligns with previous studies indicating that pericytes in the choroid partially cover the choriocapillaris^33,34^, which suggests the presence of perivascular stromal cells in our model.

RPE and EC interactions are crucial for choriocapillaris survival^35,36^. Moreover, soluble factors from RPE cells are necessary for vessel tube formation^37^. Nevertheless, in the sprouting assay, we compared the E and EF groups, which were not co-cultured with RPE cells, with the mouse choroid in which the RPE and sclera were not removed as a control, and the EF value was comparable to that of the choroid. Previous ex vivo research also did not show any difference between the presence and absence of RPE depending on the conditions^22^.

Previous studies indicate that vascular sprouts were accompanied by pericytes^22^, and the presence of pericytes on mature capillaries is necessary for endothelial stabilization and regulation of blood flow^38,39^. In this model, the extension of choroidal fibroblasts in the sprouting assay may play a role as pericytes. This sprouting assay has been performed using ex vivo samples from mice or rats^22^. Isolating the choroid from the eyes offers a direct method to comprehend in vivo findings for angiogenesis research and drug screening^40–42^. However, this approach demands a substantial number of experimental animals and considerable time for handling. In this aspect, our current data indicate that our in vitro model is highly valuable to replace and reduce animal testing and save efforts in these procedures.

To develop an imitated choroid model for studying the underlying mechanisms of choroidal abnormalities and searching for effective molecules in ocular diseases, in vitro engineered tissues have been suggested ^16–18,28^. Manian KV et al. integrated iPSC-derived cells, including RPE cells, ECs, pericytes, and mesenchymal stem cells from human donors, into a poly(ethylene glycol)-based extracellular matrix^16^. Additionally, Song MJ et al. engineered RPE–choriocapillaris structures by bioprinting endothelial cells, pericytes, and fibroblasts in poly-(lactic-co-glycolic acid) scaffold^18^.

In this study, we introduced a choroid i*n vitro* model which is easily reproducible without a need for specialized techniques such as 3D printing or biomaterial synthesis. However, vascular structures of the choroid in each layer should exhibit specific gene expressions in their vascular endothelial cells. While immunostaining indicated the formation of choriocapillaris-like vascular structures in this model, a detailed analysis has not been conducted, which necessitates future comparison analyses with human choroidal vessels. Moreover, we evaluated vascular responses of this model with vasodilator drugs. To search for new drugs that can prevent the progression of pathologic myopia by increasing choroidal blood flow, although animal models such as form-deprived or lens-induced myopia models have been used so far^15,43,44^, the in vitro model developed in this study could evaluate drug candidates by assessing vasodilation effects. Indeed, bunazosin demonstrated protective effects on myopia progression by increasing choroidal blood perfusion in mice^23^, as the results of this study indicated that vascular structures constructed in this model undergo dilation. We assume that by assessing the desired effects of drugs or stimuli on choroidal diseases, including choroidal neovascularization and CC atrophy, our choroid model can establish its validity as an experimental system for in vitro screening. However, this study had a limitation in that it did not conduct drug screening under detailed conditions. Therefore, further verification in drug screening with various conditions such as drug types, timepoints, and concentrations using this model should be conducted in future studies.

In summary, while further studies are essential to deepen our understandings, our co-culture system involving hiPSC-ECs, msCFs, and hiPSC-RPE cells offers a promising avenue to investigate choroidal development and the differentiation of choroid cells. Moreover, our study can provide a valuable platform for the screening of potential drug candidates aimed at addressing choroidal vascular diseases, encompassing conditions such as AMD and pathologic myopia. The establishment of such in vitro models not only contributes to our insights into choroidal pathophysiology but also holds great potentials for advancing therapeutic interventions in ocular diseases.

## Materials and Methods

### Animal

Mice (C57BL/6 J and BALB/c) were obtained from CLEA Japan (Tokyo, Japan). All procedures involving animals were conducted in accordance with the guidelines of the Ethics Committee on Animal Research of Keio University School of Medicine (#16,017), the ARVO Statement for the Use of Animals in Ophthalmic and Vision Research, and the International Standards of Animal Care and Use, Animal Research: Reporting in Vivo Experiments.

### Cell culture

Mouse choroidal fibroblasts (msCFs) were isolated from C57BL/6 J mouse eyes using a modified method based on the procedures outlined by Djigo AD et al.^17^ and Benedicto et al.^45^ Briefly, the RPE/choroid tissue was isolated from 6–8-week-old mouse eyes. The RPE was removed from the 0.25% trypsin/EDTA-treated tissue through vigorous pipetting. To make a single-cell suspension, the choroid tissue was incubated with 6.25 mg/ml collagenase A (038–24,561, FUJIFILM Wako Pure Chemical Corporation, Osaka, Japan), 6.25 mg/ml dispase II (17,105,041, Thermo Fisher Scientific, Sunnyvale, CA, USA), and 62.5 μg/ml DNase (EN0521, Thermo Fisher Scientific, Sunnyvale, CA, USA) solution at 37 °C for 15 min, followed by incubation in 0.25% trypsin/EDTA at 37 °C for 5 min. Endothelial cells (ECs) were removed from this dissociated cell suspension using Dynabeads CD31 (11155D, Invitrogen, Logan, UT, USA) following the manufacturer’s instructions. The suspension was cultured in Dulbecco's Modified Eagle Medium with high glucose (08,457–55, Nacalai Tesque, Kyoto, Japan) supplemented with 10% fetal bovine serum (FBS) and 1% streptomycin-penicillin (P/S) at 37 °C in a 5% CO_2_ incubator. For the choroid model, msCFs with passage 4–10, which outgrew the other cells, were utilized.

Human induced pluripotent stem cell (hiPSC)-derived ECs (iCell Endothelial Cells 11,713, FUJIFILM Cellular Dynamics, Inc., Madison, WI, USA) and RPE (iCell Retinal Pigment Epithelial 01,279, FUJIFILM Cellular Dynamics, Inc., Madison, WI, USA) cells were obtained from FUJIFILM Cellular Dynamics, Inc. (Madison, WI, USA). hiPSC-ECs were cultured on 30 µg/ml fibronectin-coated plates in VascuLife VEGF Medium (LL-0003, Lifeline Cell Technology, Walkersville, MD, USA) with iCell Endothelial Cells Medium Supplement (M1019, FUJIFILM Wako Pure Chemical Corporation, Osaka, Japan) at 37 °C in a 5% CO_2_ incubator. hiPSC-RPE cells were cultured on plates coated with 2.5 µg/ml vitronectin in MEM alpha (12,571,063, Thermo Fisher Scientific, Sunnyvale, CA, USA) supplemented with 1% N-2 Supplement (17,502,048, Thermo Fisher Scientific, Sunnyvale, CA, USA), 55 nM hydrocortisone (H6909, Sigma-Aldrich, St Louis, MO, USA), 250 µg/ml taurine (T0625, Sigma-Aldrich, St Louis, MO, USA), 14 pg/ml triiodo-L-thyronine (T5516, Sigma-Aldrich, St Louis, MO, USA), 5% FBS, and 1% P/S at 37 °C in a 5% CO_2_ incubator. Both hiPSCs with passage 3–5 were used in this study.

### Construction of the choroid in vitro model

hiPSC-RPE were cultured on a vitronectin-coated 24-well plate for two weeks and then used to construct the choroid model (Supplementary Fig. S2). hiPSC-ECs or cells containing a 1:1 mixture of hiPSC-ECs and msCFs were seeded into a fibronectin-coated transwell insert. The insert was placed in a 24-well plate with or without RPE cells, and the choroidal models were cultured with the hiPSC-EC medium for one week at 37 °C in a 5% CO_2_ incubator while replacing the medium every two days.

### Cryosection staining

For cryosectioning, 8-week-old BALB/c mouse eyes were enucleated and fixed in 4% paraformaldehyde (PFA) solution for 3 days. The constructed choroid model was then removed from the transwell insert and fixed in 4% PFA solution for 1 h. After rinsing with PBS, the samples were sequentially incubated in 10%, 20%, and 30% sucrose solutions for 1 h each and subsequently embedded in Tissue-Tek® O.C.T. Compound (Sakura Finetek USA, Inc., Torrance, CA, USA) for 1 h before being frozen in liquid nitrogen. Cryosectioned slides, 6 µm thick, were obtained using a Leica CM3050S Cryostat (Leica Microsystems, Reichert Jung, Germany) and air-dried. The slides were counterstained with hematoxylin and eosin (H&E), and images were captured using an Olympus BX53 microscope (Olympus, Tokyo, Japan).

For immunohistochemistry, the cryosectioned slides were washed for 5 min with PBS, permeabilized with 0.1% Triton X-100 for 5 min, and then blocked with 1% bovine serum albumin solution in PBS for 1 h at room temperature. Primary antibodies were applied in the blocking buffer at 4 ℃ overnight, followed by PBS washing three times (5 min per wash). Subsequently, the slides were incubated with secondary antibodies and 4’,6-diamidino-2-phenylindole (DAPI, Dojindo, Kumamoto, Japan) in PBS, washed again with PBS, and mounted on glass slides using a mounting medium. Finally, the samples were observed under a confocal laser-scanning microscope (Olympus FV3000, Tokyo, Japan). The primary antibodies were Armenian hamster anti-CD31 (ab119341, Abcam, Cambridge, MA, USA), rat anti-endomucin (MAB2624, Millipore, Billerica, MA, USA), rabbit anti-PDGFRβ (3169S, Cell Signaling Technology, Denvers, MA, USA), mouse anti-alpha smooth muscle actin (14–9760-82, Invitrogen, Logan, UT, USA), rabbit anti-lumican (ab168348, Abcam, Cambridge, MA, USA), rat anti-perlecan (sc-33707, Santacruz, Dallas, TX, USA), rabbit anti-collagen I (SAB4500362, Sigma-Aldrich, St Louis, MO, USA), rabbit anti-collagen III (ab184993, Abcam, Cambridge, MA, USA), and rabbit anti-collagen IV (ab6586, Abcam, Cambridge, MA, USA). The secondary antibodies were Alexa Fluor 488-conjugated anti-Armenian hamster (ab173003, Abcam, Cambridge, MA, USA), Alexa Fluor 488-conjugated anti-rabbit (A21206, Invitrogen, Logan, UT, USA), Alexa Fluor 488-conjugated anti-rat (A21208, Invitrogen, Logan, UT, USA), Alexa Fluor 555-conjugated anti-mouse (A31570, Invitrogen, Logan, UT, USA), and Alexa Fluor 555-conjugated anti-rabbit (A31572, Invitrogen, Logan, UT, USA).

### Quantification of choroidal vessel formation

For the evaluation of vessel formation, the vessels in choroid tissues isolated from 8-week-old BALB/c mouse eye and the constructed choroid models were immunostained with CD31 antibody. Briefly, the samples were fixed for 45 min at room temperature in 4% PFA solution and washed with PBS. To facilitate clear observation of choroid vessels by removing RPE pigments, the choroid tissues were treated with a melanin bleach kit (24,883–1, Polysciences, Warrington, PA, USA), following the manufacturer's instructions. Subsequently, the samples were blocked in a 1% bovine serum albumin solution in PBS containing 0.5% Triton X-100 for 1 h. The primary antibody (Armenian hamster anti-CD31, ab119341, Abcam, Cambridge, MA, USA) was applied in blocking buffer at 4 °C for two overnights, followed by three washes in PBST and incubation with the secondary antibody (Alexa Fluor 488-conjugated anti-Armenian hamster, ab173003, Abcam, Cambridge, MA, USA). Finally, the samples were washed in PBST and mounted using a mounting medium. Stained images of samples were acquired using a confocal laser-scanning microscope (Olympus FV3000, Tokyo, Japan). The CC layer of the mouse choroid within 500 to 1000 μm around the optic nerve was captured. Quantitative analyses of vessel area, total number of junctions, vessel length, and lacunarity of obtained images were analyzed by AngioTool software (National Institutes of Health, National Cancer Institute)^46^. The average value of 4 to 5 images obtained from one sample was treated as one value.

### Sprouting assay

Sprouting assays were performed as described by Shao Z et al.^22^ 8-week-old C57BL/6 J mouse eyes were enucleated, and unwanted tissues were removed, which leaves only the RPE, choroid, and sclera. The central regions of the choroid tissue, along with RPE and sclera, was then cut into approximately 1 mm × 1 mm fragments. The mouse choroid fragments and the constructed choroid models (E and EF), removed from the transwell insert, were embedded in 100 µL of Matrigel matrix (354,234, Corning, NY, USA). The mixture was incubated at 37 °C in a 5% CO_2_ incubator for 10 min, followed by adding the hiPSC-EC medium. After 5 days of culture, images of the samples were captured using an Olympus BX53 microscope (Olympus, Tokyo, Japan). The areas of sprouting were quantified using ImageJ software (National Institute of Health).

### Quantitative PCR (qPCR)

Total RNA was extracted from the constructed choroid models (EF and EF/R) and 6–8-week-old C57BL/6 J mouse choroid tissues using TRI reagent (TR118, MRC Global, Cincinnati, OH, USA) following the manufacturer's protocol. Subsequently, reverse transcription was performed to obtain cDNA using ReverTra Ace qPCR RT Master Mix (FSQ-301, TOYOBO, Osaka, Japan). qPCR was performed using THUNDERBIRD SYBR qPCR Mix (QPS-201, TOYOBO, Osaka, Japan), and PCR amplification was performed using Applied Biosystems 7500 Fast PCR System (Applied Biosystems, Waltham, MA, USA). The primer sequences used in this study are listed in Table 1. To quantify differential gene expression, the ΔΔCT method was employed, and the results were normalized to the reference gene (*β-actin*).

**Table 1:** Primer list.

Name	Direction	Sequence (5′ → 3′)	Accession Number
*β-actin*	Forward	GGAGGAAGAGGATGCGGCA	NM_007393.5
	Reverse	GAAGCTGTGCTATGTTGCTCTA	
*Acta2*	Forward	GTACCACCATGTACCCAGGC	NM_007392.3
	Reverse	GCTGGAAGGTAGACAGCGAA	
*Cspg4*	Forward	TAGGGAGCAGGCAAACGAAG	NM_139001.2
	Reverse	AAACTCAAACGACGCACAGC	
*Col1a1*	Forward	TTCTCCTGGCAAAGACGGAC	NM_007742.4
	Reverse	CGGCCACCATCTTGAGACTT	
*Pdgfra*	Forward	CGGAACCTCAGAGAGAATCGG	NM_001083316.2
	Reverse	TCCCCATAGCTCCTGAGACC	
***Pdgfrb***	Forward	TGTGCAGTTGCCTTACGACT	NM_001146268.1
	Reverse	CGCTACTTCTGGCTGTCGAT	

### Drug treatment

The α1-adrenergic blocker, bunazosin hydrochloride (B689585; Toronto Research Chemicals Inc., ON, Canada), known as a vasodilator^47^, and the α1-adrenergic receptor agonist, phenylephrine hydrochloride (P0398, TCI chemicals, Tokyo, Japan), known as a vasoconstrictor^24^, were individually dissolved in PBS to a concentration of 10 mM. Each solution was then adjusted to 10 μM in the hiPSC-EC medium. The medium was replaced in the constructed model (EF/R) with a medium supplemented with each reagent. After 5 h of incubation, the expected effect of each vasodilator or vasoconstrictor on the constructed model was evaluated using the method for quantification of choroidal vessel formation as described above.

### Statistical analysis

The results of this study are presented as the mean ± standard deviation. Statistical analysis was conducted using one-way ANOVA, followed by a post-hoc Tukey’s test. Statistical significance was determined when the p-value was less than 0.05.

### Supplementary Information

Supplementary Figures.

## Data Availability

The datasets used during the current study are available from the corresponding author on reasonable request.
