# Supplementary material for: Establishment of an in vitro choroid complex system for vascular response screening
Source: Sci Rep. 2024 Jul 12;14:16129. doi: 10.1038/s41598-024-67069-8 (PMC11245503; doi:10.1038/s41598-024-67069-8)
Supplement: Supplementary file 1 — Supplementary Figures. [file 41598_2024_67069_MOESM1_ESM.docx]

**Supplementary information**


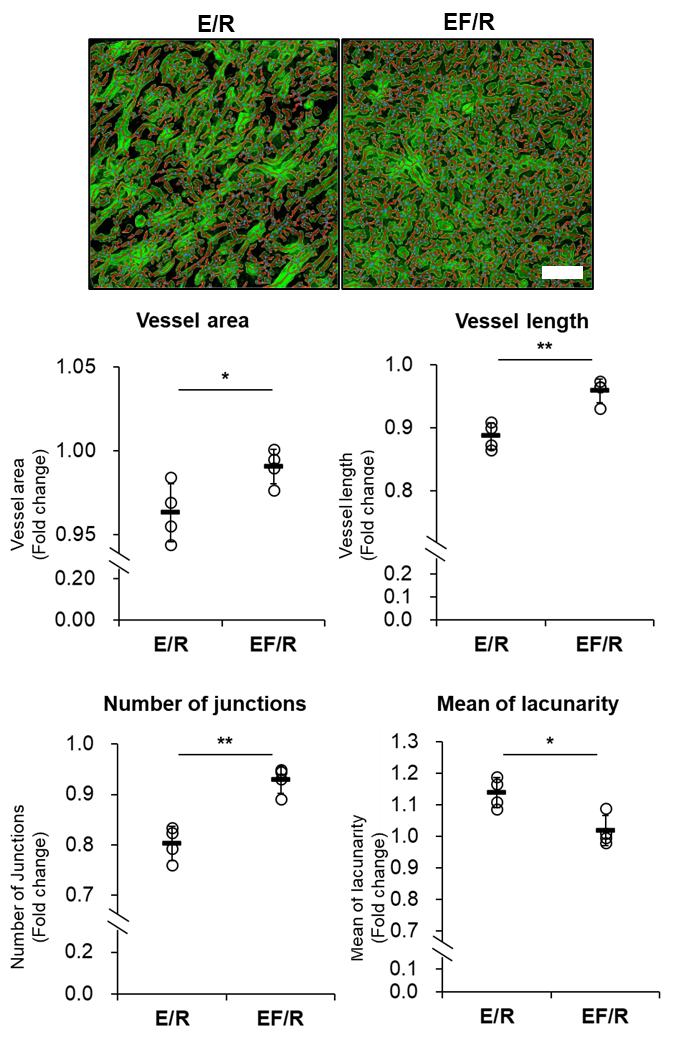


Supplementary Fig. S1. Quantification of vessel formation compared the models with (EF/R) and without (E/R) co-culture of choroidal fibroblasts. Segmented and skeletonized immunofluorescence images and analysis of vessel area, vessel length, number of junctions, and mean of lacunarity using AngioTool. The quantification data was normalized by the values of the mouse choroid. Scale bar: 100 µm. n = 4. * p < 0.05, ** p < 0.01, two groups were tested with Student’s t-tests.

**
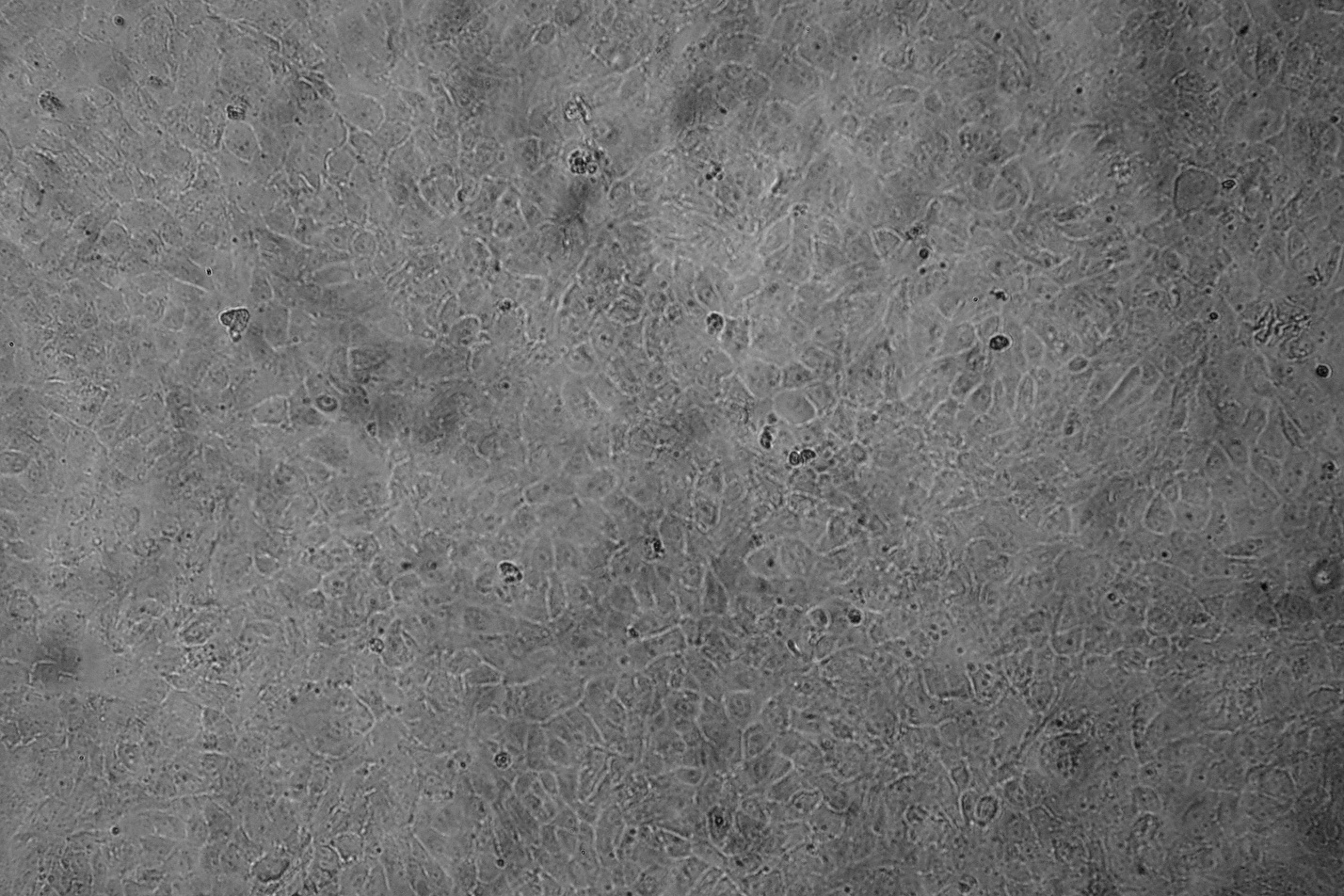
**

Supplementary Fig. S2. Microscope image of hiPSC-RPE cells after 2 weeks of culture. RPE cells became typical hexagonal shape.
